# Acquiring Financial Support for Children’s Sports Participation: Co-Creating a Socially Safe Environment for Parents from Low-Income Families

**DOI:** 10.3390/children10050872

**Published:** 2023-05-12

**Authors:** Lonneke van Leeuwen, Angelique Ruiter, Kirsten Visser, Heidi M. B. Lesscher, Merel Jonker

**Affiliations:** 1 Public Health Department, Julius Center for Health Sciences and Primary Care, University Medical Center Utrecht, 3584 CG Utrecht, The Netherlands; 2 Department of CoDesign, Utrecht University of Applied Sciences, 3584 CH Utrecht, The Netherlands; angelique.ruiter@hu.nl; 3 Department of Human Geography and Spatial Planning, Faculty of Geosciences, Utrecht University, 3584 CB Utrecht, The Netherlands; k.visser@uu.nl; 4 Department of Population Health Sciences, Division of Behavioural Neuroscience, Faculty of Veterinary Medicine, Utrecht University, 3584 CS Utrecht, The Netherlands; 5 Faculty of Law, Economics and Governance Utrecht, Utrecht School of Law, Utrecht University, 3512 BK Utrecht, The Netherlands; m.jonker@uu.nl

**Keywords:** club-organized sports, children, low-income, parents, social safety, co-creation

## Abstract

Despite the many benefits of club-organized sports participation for children, sports participation is lower among children from low-income families than among those from middle- or high-income families. Social safety experienced by parents from low-income families is an important facilitator for parents to request financial support for their children’s sports participation. Therefore, the first aim of this study was to better understand parental social (un)safety in the context of acquiring financial support for children’s sports participation and how to create a safe social environment for low-income parents to request and receive this financial support. The second aim was to describe the co-creation process, which was organized to contribute to social safety solutions. To reach these goals, we applied a participatory action research method in the form of four co-creation sessions with professionals and an expert-by-experience, as well as a group interview with parents from low-income families. The data analysis included a thematic analysis of the qualitative data. The results showed that from the perspective of parents, social safety encompassed various aspects such as understandable information, procedures based on trust, and efficient referral processes. Sport clubs were identified as the primary source of information for parents. Regarding the co-creation process, the study found that stakeholders tended to overestimate parental social safety levels. Although the stakeholders enjoyed and learned from the sessions, differences in prior knowledge and a lack of a shared perspective on the purpose of the sessions made it challenging to collaboratively create solutions. The study’s recommendations include strategies for increasing parental social safety and facilitating more effective co-creation processes. The findings of this study can be used to inform the development of interventions that contribute to a social environment in which parents from low-income families feel safe to request and receive financial support for their children’s sports participation.

## 1. Introduction

It is widely known that children benefit from club-organized sports participation (hereafter: sports participation), such that sports participation contributes to children’s physical and mental health [1,2]. Psychological benefits of sports participation for children include emotional self-efficacy and fewer depressive symptoms, as well as social integration [3]. As such, sports participation is considered a tool for contributing to children’s health, societal participation, and social inclusion [4]. Especially for children from underprivileged groups, sports participation is believed to result in social advantages, both on a personal and a societal level. However, studies in several countries have shown that sports participation is lower among children from low-income families compared to those from middle- or high-income families (e.g., Refs. [4,5,6]). As such, sports clubs seem to be less accessible to children, who may benefit most from sports participation.

One potential explanation for this difference is that parents from low-income families experience multiple barriers to organizing their children’s sports participation. For instance, parents report issues with time and scheduling [7,8,9,10], transportation [7,10], and acquiring, processing, and providing necessary information for organizing sports participation [10]. They also experience financial barriers, such as the cost of sports participation [7,8,10,11]. To mitigate this financial barrier, many municipalities or countries have implemented fee assistance programs to financially support low-income families and children in their participation in sports and other recreational activities [8,12,13,14]. Such programs are considered promising as they help families reduce the costs associated with sports participation, thereby enabling children from low-income families to access opportunities for sports participation [13].

However, accessing these programs may be challenging for parents from low-income families. Studies show that parents may be unaware of available fee assistance programs [13,15,16]. Even when parents are aware of such programs, they encounter barriers when trying to access them. For example, an interview study with parents who utilized a fee assistance program [13] showed that parents experienced trouble understanding the documentation requirements, experienced shame for having to request help and enroll in a fee assistance program, and were treated negatively, which they perceived as being due to their enrollment in the program. Another interview study [17] also showed that parents experienced shame. They felt that they were unable to meet their own and others’ expectations regarding their ability to afford sports independently. Their pride suffered because they assumed that others would perceive them as less worthy. Having to expose their financial situation in order to obtain financial support made them feel extra vulnerable. 

The lengthy and dehumanizing application and administrative processes that parents from low-income families must go through to obtain financial support for their children’s sports and leisure activities are often considered exclusionary [18] and trigger experiences of stigma and isolation [12,19]. Furthermore, shame can arise because parents may perceive seeking financial support as a failure to meet societal standards [20]. Social Safety Theory suggests that maintaining social bonds is a critical aspect of human behavior, and threats to social safety can cause psychological stressors such as shame [21]. Social safety is characterized by social acceptance, affiliation, belonging, and connection, while social threat is characterized by conflict, aggression, discrimination, and exclusion. Beliefs about social safety shape thoughts, emotions, how individuals navigate their social worlds, and the types of relationships they develop. As individuals are embedded in socio-ecological systems, social safety may include interactions/bonds on multiple social environmental levels ranging from more proximal (e.g., friend, family) to distal (e.g., country, world), with levels such as community, neighborhood, and city in between [21].

Perceptions related to social unsafety may explain why in the Netherlands, fee assistance programs are underutilized (personal communication with support organizations, 2020) and why the benefits of sports may be out of reach for those facing social problems [22]. It has been argued that this gap needs to be bridged [15] and that lengthy, burdensome, and shame-triggering application processes should be avoided [12].

This motivated us to conduct this qualitative study and to include co-creation research with stakeholders. Co-creation is the collaborative process of stakeholders working together to develop innovative ideas and policies that reflect citizens’ needs, achieve democratic decision-making, and purposefully engage diverse groups [23,24]. It involves a focus on the interactions between stakeholders (e.g., Refs. [25,26,27]), empowering them to feel ownership of complex problems, and giving them the tools to act [28,29]. The facilitation, organization, and participation of co-creation involve five elements: purpose, formality, ownership, motivation, and places/spaces [23] (see Table 1 for more details). However, there is still a lack of understanding regarding how co-creation is practiced in real-world settings [23] and the prerequisites that facilitate successful co-creation [30].

### Aims of the Study

To summarize, interventions that are implemented to increase sports participation by children from low-income families may take the form of fee assistance programs. However, such programs may be underutilized because of shame and perceived social unsafety. The first aim of the study is to better understand parental social (un)safety in the context of acquiring financial support for children’s sports participation and how a social environment for parents from low-income families can be created in which parents feel safe to request and receive financial support for their children’s sports participation. 

In order to address the first aim of how to create a safe social environment for low-income families, a co-creation process with stakeholders was organized. The second aim of the study is to describe this co-creation process, which consisted of the following elements: (1) purpose; (2) formality; (3) ownership; (4) motivation and incentives; and (5) place and space [23].

As such, the first aim focuses on the *results* of the co-creation sessions in terms of a better understanding of social (un)safety in the context of organizing children’s sports participation. The second aim focuses on understanding the *process* of co-creation.

## 2. Materials and Methods

### 2.1. Study Context

The study took place in Utrecht, a city in the middle of the Netherlands. In Utrecht, low-income residents can autonomously apply for a U-pass (city pass) with credit for recreation and societal participation, including their children’s sports participation. Once parents have a city pass, they can apply to Paul Verweel Sportfonds (Paul Verweel Sports Fund) for a voucher to purchase sports accessories for their children. If these budgets are inadequate, parents or intermediaries such as neighborhood team members, youth workers, or teachers can apply at two other national foundations (Stichting Leergeld and Kinderhulp).

Four stakeholder co-creation sessions focusing on social safety were organized by the researchers, between September and December 2022. Three sessions were situated in community centers; one session was situated in a room in the office space of one of the participants’ institutions. Participants were local stakeholders and mostly professionals (see Section 2.3. for more information).

The stakeholder co-creation sessions were considered a logical follow-up step after the Vital@2040 study [10], which identified barriers to sports participation in Utrecht. The study results were presented to the professionals and parents who participated in the study, and the group discussed which barriers they prioritized and thought should be acted upon. One of the priorities identified was creating a socially safe environment for parents to obtain financial support for their children’s sports participation in a non-complex manner without experiencing situations that trigger shame. As a result, social safety became the focus of the stakeholder co-creation sessions.

Additionally, after the second stakeholder co-creation session, a group interview with only parents from low-income families was organized in a café in the center of Utrecht. The goal of this group interview was to obtain the perspective of parents on the output of the first and second stakeholder co-creation sessions and their input for the third and fourth sessions. The group interview was set up in accordance with parents’ preferences to share their experiences and perspectives in a smaller group with only peers and in an informal setting, with a researcher present and, if needed, one professional. After attending the group interview, the participants were given a gift card from a supermarket worth €15.

### 2.2. Design

Qualitative research, which focuses on exploring how and why questions, is particularly suited to our aim of gaining an in-depth understanding of parental social (un)safety in the context of acquiring financial support for children’s sports participation and how to create a social environment for parents from low-income families [35]. In this study, we conducted stakeholder co-creation sessions and a parental group interview to gather qualitative data.

#### 2.2.1. Stakeholder Co-Creation Sessions

Co-creation research, as a participatory action research method, involves stakeholders, including researchers, working together to set objectives, gather data, analyze the results, and develop solutions [36,37]. The aim is to align the interests of the participating stakeholders and make the research process as inclusive as possible [38].

During the 2-h stakeholder co-creation sessions, qualitative data were gathered through:

1. observations (field notes);
2. asking reflexive questions or sharing observations;
3. canvases to invite participants to provide input and to structure their input using co-design as part of the Participatory Action Research (PAR) approach;
4. an evaluation form collecting participants’ feedback on the sessions.

The researchers prepared the sessions, and the activities and working methods for sessions 2–4 were chosen based on the discussions and voiced preferences during the previous sessions. Each session, we brought our reflections back to the stakeholders to analyze the outcome together. We aimed to generate potential solutions and plans for future experiments by the end of the four sessions. An outline of the sessions is provided in Table 2. More detailed information about the activities is provided in Section 2.5.

#### 2.2.2. Parental Group Interview

During the one-hour interview, parents were updated on the output of the first two stakeholder co-creation sessions, and a number of prepared questions were posed about the output.

### 2.3. Participants

Participants for both the stakeholder co-creation sessions and the parental group interview were selected using a convenience sampling approach. Specifically, the individuals were drawn from the researcher’s network, and many had also participated in the prior Vital@2040 study.

#### 2.3.1. Stakeholder Co-Creation Sessions

The participants were all working in the city of Utrecht. Thirteen individuals expressed interest and availability for the sessions. These individuals worked at organizations that parents can turn to for financial support for children’s sports participation, organizations for social work or financial support, organizations supporting youth in physical activity, a sports club, a policy maker, a knowledge center for sport and physical activity, and an expert-by-experience as a representative of low-income parents. Unfortunately, not all individuals who registered were present during all four sessions, and two organizations (including the sports club) were not able to attend any of the sessions. The number of participants per session ranged from six to eight.

#### 2.3.2. Parental Group Interview

The participants were three parents from low-income families in Utrecht. One professional participant from the co-creation sessions attended the interview to provide a summary of the co-creation session outputs.

### 2.4. Procedure

#### 2.4.1. Stakeholder Co-Creation Sessions

Potential participants were informed about the co-creation sessions and invited by email. Interested individuals could provide their availability for the suggested dates for the sessions. Those interested received a follow-up email with the dates and information about the study, including its purpose, the data to be collected, and a statement that attending the sessions meant that participants agreed that their input would be collected, stored, and used for the study. The email also included information about how the researchers dealt with data privacy.

#### 2.4.2. Parental Group Interview 

Potential participants were informed about the interview/study and were invited by telephone. Interested parents provided their availability for the suggested dates for the sessions. A date was chosen on which all interested parents could attend. At the start of the interview, parents were informed about the purpose of the study and that the researcher would make anonymous notes without any direct or indirect identifying information. It was stated that attending the interview meant that parents agreed that their input would be collected, stored, and used for this study.

### 2.5. Materials

#### 2.5.1. Stakeholder Co-Creation Sessions

***Canvas ‘Future Probing’ (Session 1).*** With a prepared paper canvas, participants reflected on the following questions: (a) What are the desired future outcomes in relation to social safety that will support parents from low-income families in arranging sports participation for their children? (b) Who needs to change what to achieve this desired outcome? (c) How can this be achieved?

***Canvas ‘Scenario-Based Action Plan’ (Session 2)*.** The input on the canvas ‘Future Probing’ was used to co-create a more detailed action plan based on a scenario of a parent from a low-income family in need of financial support to organize their child’s sports participation. The canvas posed the following questions: (a) Where in the process may a parent need support? (b) Which professional or volunteer has to or is able to provide this support (intermediaries)? (c) What actions are required for intermediaries to offer this support? (d) Which actions are most feasible and relevant?

***Canvas ‘Setting Up an Experiment’ (Session 4)*.** Participants co-created an action plan for an experiment they might work on in the future. This canvas asked participants to determine the goal of the experiment (e.g., gaining knowledge or desired behavior change), the design and approach of the experiment, and the baseline conditions needed for the experiment to succeed.

***Field notes*.** The researchers recorded their observations and reflections on both the process of the co-creation sessions and the topic of social safety.

***Reflective questions*.** Each session started with the researchers reflecting on the content and process of the previous session, which was used as input for the following sessions in our Participatory Action Research (PAR) approach. After presenting this reflection, participants were asked whether they recognized the observation and if there was anything that needed to be addressed at that moment. In line with the method of responsive evaluation [39], the researchers posed questions to understand how participants experienced the process, their concerns, and any issues, and their ideas for the following co-creation session.

***Evaluation*.** After the fourth session, participants received an email containing a link to an anonymous online evaluation form with the following questions: (a) How many sessions did you attend? (response choices: 1–4 sessions); (b) How would you rate the co-creation sessions? (response choices: 1–5 stars); (c) What, in your opinion, was positive about the sessions? (d) In which areas can the sessions be improved? (e) Did you personally gain anything from the sessions, and if so, what did you gain?

#### 2.5.2. Parental Group Interview

***Field notes*.** The researcher recorded her observations and reflections on the topic of social safety.

***Questions.*** During the interview, the output of the stakeholder co-creation sessions was presented, and participants were invited to provide their perspective on this output. They were asked, for example, how they would describe a safe and supportive conversation about barriers to their children’s sports participation with a professional or volunteer, or where they would turn for support.

### 2.6. Data Analysis

#### 2.6.1. Socially Safe Environment

The field notes and canvases of the stakeholder co-creation sessions and the field notes of the parental group interview underwent coding using a thematic analysis approach [40]. This means that the data were first read multiple times for familiarization. Then broad categories of data were formed based on the topics of the stakeholder canvases, including the discussions that took place as a result of these canvases and the questions that were asked during the parental group interview (e.g., what are characteristics of social safety or a socially safe environment?). Relevant pieces of data from both the stakeholder group and the parental group were categorized in these broad categories, and within categories we searched for related data that would form themes. This approach also enabled us to identify any differences in the social safety perspectives of the stakeholders and the parents. A naturalistic research model was used, meaning that the data were treated as if they gave direct access to participants’ experiences. The naturalism research model differs from, for example, a constructionism research model, which focuses on understanding how social realities are constructed and sustained [35].

#### 2.6.2. Elements of Co-Creation

The researchers examined the categories as defined by Puerari et al. (2018) [23] (see Table 1) to analyze how these dynamics existed in the setting of our research. The elements of co-creation were analyzed by studying the field notes and evaluations of the stakeholder co-creation sessions. Similarly, a thematic analysis approach [40] was employed, where the researchers independently categorized their observations, insights, and evaluations according to each element of co-creation, comparing and discussing their findings until consensus was achieved.

## 3. Results

The results are presented in two parts. First, the perspectives of parents and professionals about socially (un)safe environments are discussed. Second, the stakeholder co-creation process for each of the five co-creation elements is described.

### 3.1. Perspectives of Parents and Professionals about a Socially Safe Environment

The thematic analysis of the data collected during the co-creation sessions and the parental group interview showed that discussions and output on the canvases mainly focused on the following three topics: (1) characteristics of social safety or a socially safe environment; (2) sources of information and support; and (3) future interventions.

#### 3.1.1. Characteristics of Social Safety or a Socially Safe Environment

Both the stakeholder group and parents discussed what constitutes social safety or a socially safe environment for parents to arrange financial support. Identified themes included the interaction between parents and intermediaries, the process of providing or acquiring financial support, the lack of trust implied by policy and procedures, and social norms.

**Interaction between parents and intermediaries.** Parents described that in a socially safe environment, intermediaries display a sincere interest in the parents’ request or situation (e.g., by making notes), demonstrate an understanding of how hard it is for parents to converse about their (financial) challenges, and exhibit a proactive and persevering attitude as well as experience in providing support until the request is finalized or the issue is resolved. The stakeholder group added that intermediaries should be able to identify when parents may not directly ask for support but may need it. Parents further described that the interaction with intermediaries should be focused only on providing support for arranging sports participation for their children and not on other challenges parents could potentially experience. As one parent described it, “I do not have a problem; I just want to utilize the financial support options for my children’s sports participation”. The subsequent discussion revealed that this desire stems from a distrust of and negative experience with authorities and institutions. They expressed a lack of trust that that sharing information about their challenges would not lead to negative consequences in the future. They explained that they wish to live ‘under the radar’ and prevent file formation as much as possible. This suggests that, for these parents, social safety also includes professionals and organizations conveying that information, which is not shared with other organizations or used for other purposes.

**The process of providing or acquiring financial support.** The stakeholder group described how, in a socially safe environment, parents know where to turn for information and financial support. The parent group added that the information they acquire (e.g., about options for financial support) is understandable for parents. As a positive example, they described the initiative of Stichting Leergeld, where parents only had to fill out a simple form in order to obtain a voucher for a winter coat for their children.

In addition to being understandable, information should be consistent. Thus, regardless of which organization or professional a parent turns to for information, the information should be the same. Furthermore, both the stakeholder and parent groups described that the process between a parent’s implicit request for financial support and actually receiving the support should include as few referrals as possible. This arises from the fact that parents may feel ashamed every time they have to explain their need for financial support to someone. One parent, for example, described how she went to a sports club to register her child there. She requested the sports club use the budget on her city pass. To her, this request was a moment during which she felt ashamed. However, the person at the sports club did not know how to arrange that, and so the parent was asked to come back later. Later, she again had to explain her situation to another person and again experienced shame. This particular parent was highly motivated to arrange her child’s sports participation, so she chose to persevere despite experiencing shame repeatedly. In line with this, the stakeholder group opined that the first professional or volunteer that a parent turns to for financial support should be able to assist at least to some extent or should ensure that a parent experiences a cordial transfer to another professional, volunteer, or organization.

**(Dis)trust implied by policy and procedures.** Both the stakeholder and parent groups expressed that the policy and procedures to obtain financial support should express trust rather than distrust. For example, parents feel that they have to submit a lot of personal financial information to prove their eligibility for financial support. This perception leads them to feel that the organizations providing support do not trust them

**Social norms.** Parents expressed that if they knew other parents also face financial challenges in arranging their children’s sports participation and utilize available support options, it would reduce their sense of shame. This suggests that supportive social norms would contribute to a sense of social safety.

#### 3.1.2. Sources of Information and Support

According to the stakeholder group, parents tend to seek support from various organizations or (semi-)professionals when they encounter financial barriers to sports participation: neighborhood teams, funds, social work, youth work, education, general practitioners, youth health care, community centers, and the sports club. The parents, however, explained that they only use(d) the sports club as the entry point for information about how financial support could be arranged. The main reason was that they had no experience or knowledge about how each of the other organizations would be able to support them in arranging their children’s sports organization. Parents perceived all these organizations as ‘institutions’ and felt hesitant to seek support from them or share their challenges openly. For them, the sports club feels like a logical and safe entry point. Although not explicitly mentioned by the parents, it is worth noting that other parents also serve as a source of information and support. In relation to the previously mentioned initiative of Stichting Leergeld, parents explained that communication about the winter coat initiative was easily shared among their social network of peers via texts. During the group interview, parents shared their experiences in acquiring financial support and offered advice to each other on overcoming barriers. For example, one parent explained to another parent how she was able to utilize the city pass to finance her child’s costs for public transportation. The other parent thanked her for this advice, as this was new information to her.

#### 3.1.3. Future Interventions

Not surprisingly, most of the potential future interventions that were mentioned were aimed at increasing the characteristics of social safety. In relation to intermediaries’ social safety behaviors, interventions should focus on increasing the knowledge and skills necessary to be able to understand, communicate, support, or cordially transfer parents to sources for (financial) support. To promote social safety behaviors among intermediaries, it is important to establish preconditions, such as allocating sufficient time for them to fulfill their tasks. 

To improve the process of referring parents to other professionals, volunteers, or organizations to obtain financial support, the stakeholder group proposed appointing a dedicated intermediary who can support and monitor parents in navigating financial barriers to sports participation, aiming to reduce the dropout rate during referral processes. Furthermore, the stakeholder group expected that referrals of parents to and from their respective organizations could be organized more efficiently if they collaborated more closely and were more accessible. Parents further suggested that all sports clubs should establish a relationship or collaboration with the organization providing financial support, enabling the smooth utilization of these budgets across all sports clubs. Last, parents suggested the idea of implementing an accessible counter occupied by (semi-)professionals who are able to support parents and provide information.

To reduce instances in which parents may experience shame and distrust, parents suggested that procedures for acquiring financial support should be organized more effectively. For example, they suggested that parents only need to provide relevant financial information once and that this information should be stored centrally. If organizations need this information from parents, parents can provide organizations with a link or QR code to this information.

Other future directions include supporting sports clubs in increasing their inclusiveness, developing communication campaigns that promote the idea that facing barriers is normal, utilizing financial support for children’s sports organizations, and deploying user-friendly information flyers in locations regularly accessed by parents, such as schools.

### 3.2. Elements of Co-Creation

While the preceding sections focused on the outcomes of the co-creation sessions regarding social (un)safety in the context of children’s sports participation, the subsequent sections concentrate on elucidating the co-creation process.

#### 3.2.1. Purpose

The purpose of the co-creation sessions was to bring together stakeholders and collaboratively work towards solutions that foster a socially safe environment for low-income parents to obtain financial support for their children’s sports participation. Several design canvases were used to create concrete solutions and action plans. The sessions were more focused on making than learning together, but participants indicated in the evaluation that they had learned much from exchanging with other organizations for their own work.

At the start of the first stakeholder co-creation session, participants were asked what they hoped to achieve after the sessions. They voiced different achievements, including better collaboration between the participants’ respective organizations, higher social safety for parents to express their concerns without feeling embarrassed, identification of opportunities for change/solutions, learning about the experiences of parents and the practices of professionals, knowledge derived from scientific research, practical expertise, and lived experiences being brought into the sessions, and taking concrete actions. One participant expressed a cautious attitude, stating that her future participation would depend on the progress and outcomes of the sessions.

One observation especially contributed to the sense of differing views on purpose. One participant requested that part of the coming session be devoted to collecting the other participants’ views about an information leaflet that her organization produced about local opportunities to obtain financial support for sports. Although this may be perceived as a co-creation activity and the purpose of the leaflet fit the topic of the co-creation sessions, the idea of producing the leaflet and its content were not co-created by the group. The participant appeared to leverage the session and the presence of other participants to gather expert opinions to enhance her organization’s leaflet.

Throughout the sessions, observations indicated that for some participants, the purpose was still unclear or unmet. For example, one participant repeatedly asked the researchers what the purpose of the sessions was or expressed a desire for the sessions to result in more actionable steps and opportunities for experimentation. The open approach to what should be achieved by the co-creation sessions did not seem to match her expectations or perceived purpose. Additionally, evaluation responses indicated that for a few participants, it was unclear at the beginning what they were working towards. Others did not express that the purpose was unclear or unmet. This may indicate that the co-creation sessions met their expectations, but it is also possible that these participants were more comfortable with a lack of clarity.

The different views on the purpose of the co-creation sessions may have been due to the participants’ varying prior knowledge about the barriers to facilitating sports participation for parents and professionals. Some participants had already been involved in the Vital@2040 study, which aimed to describe these barriers, and reducing them was one of the goals of their profession. Consequently, they had already formed ideas about potential solutions, and thus, they were well-informed about the currently experienced barriers and were ready to co-create and experiment with solutions. However, other participants were relatively new to the topic and were not as informed about barriers or potential solutions. These participants were more focused on learning about the problem than co-creating solutions.

Perhaps there was not enough time and attention given to building the conditions for co-creation and creating a shared perspective on the purpose of the sessions. The participants came from diverse organizations, employed varying terminologies, and focused on different aspects related to the problem, posing challenges in collaboratively generating solutions as a cohesive group. The conversations often spiraled away from working on the canvas as a group towards different viewpoints of organizations, anecdotal information, counter-arguing others’ viewpoints, and follow-up questions that went beyond the scope of the co-creation sessions. These issues were also recognized by the participants themselves, and efforts were made to enhance the sessions’ focus on generating practical solutions and fostering actionable outcomes.

#### 3.2.2. Formality

The co-creation sessions were both formal and informal. Participants were selected and invited by the researchers based on their motivation to work on the topic and their professional involvement with sports and/or low-income families. In addition, sessions were planned, and activities during the co-creation sessions were prepared upfront, which made them formal. However, the sessions were also constructed in an open, emergent way of working, allowing the methods used and content of the sessions to adapt to the issues and concerns of the participants as they went along. We observed a relaxed atmosphere where participants actively exchanged ideas and strategies from their respective viewpoints. However, for us as researchers, the informal co-creation process posed some challenges. For example, because participants needed more time to understand each other’s perspectives, the program of the co-creation sessions needed to be adjusted.

After two sessions characterized by an open, emergent way of working, the participants did not seem to have established a clear sense of purpose as a group. Some participants found it difficult to work with the informal and open emergent way of working and expressed their desire to know the goal and how to reach it. Therefore, we presented the participants with four concrete purpose options to work towards during the last two sessions, based on the ideas that they expressed previously. The stakeholders unanimously agreed to explore potential entry points for interventions and experiments to increase social safety when a parent turns to a sports club for support. At this point, participants seemed willing to take ownership of creating an experiment, as they were expressing ideas about interventions and experiments immediately.

The formal aspects of the co-creation sessions (them being invited, planned sessions, and prepared canvases) may have created expectations that the co-creation process would be linear with a clear end result. Reflecting on the (active) role of the participants and challenging them to come up with ideas helped to mitigate such expectations.

#### 3.2.3. Ownership

While we, as researchers, initiated the co-creation process, our intention was to transfer the sense of ownership to the group members who actively work in this domain. They possess the expertise to drive the project forward and formulate plans for future development. However, throughout the sessions, we noticed that some participants continued to see us as the leaders of the session, which was confirmed upon sharing this observation. We explicitly mentioned that we, as researchers, did not see our role as taking the lead in the process and creating solutions. Other participants looked at the policymakers as the owners of the problem, given their responsibility for the policy rules surrounding sports and finances for low-income households.

Indeed, in the evaluations, some participants expressed a lack of ownership, with some expecting someone to take the lead. It remained unclear, however, whether these participants meant leadership during the co-creation sessions or leadership in implementing actions and solutions. Participants did mention that part of leadership involved taking care of the agenda for the sessions and arranging a space to meet, which was what the researchers did. However, participants acknowledged that leading in terms of developing solution content was not within their area of expertise.

After reflecting on ownership during the session, there was a change in ownership for at least one participant who took the lead during the upcoming sessions. However, a few participants did not take ownership at all, including one participant who, up until the last session, could not find a way to work with the relative uncertainty of a co-creation process. They mentioned that they “normally work with strict goal setting and working towards set goals in a structured way”, indicating that this person was unfamiliar and perhaps uncomfortable with the researchers’ way of working.

One factor that may have hindered participants from taking ownership was their busy jobs, which did not allow for much time and space to take up extra activities. For example, during one session, participants agreed to do a homework task, but little input resulted from it. Furthermore, not all participants were present during all sessions. Although the researchers summarized the steps that were taken before each session, it may have been difficult for participants to feel full ownership of the process and the creation of solutions.

Lastly, the majority of participants expressed in their evaluations that reflecting on ownership helped them maintain continuity throughout the sessions and avoid getting lost in their individual approaches.

#### 3.2.4. Motivations and Incentives for Co-Creation

Participants in the co-creation sessions were primarily motivated by extrinsic factors, driven by their perceived purpose to remove barriers faced by parents from low-income families when enrolling their children in sports activities. Another motivation for participating was to further cooperation between the different organizations, which also seemed to be an extrinsic motivation born from the need to streamline processes and communication with parents (the wish to reach a desired outcome). The benefits for both parents and the organizations were clear to participants. These were lifting barriers for parents by gaining access to more information and more efficient processes.

Participants expressed in their evaluations that they liked the exchange of ideas and information, but there was limited shared ownership of the process. However, there was extrinsic motivation from having a shared goal, which was extensively discussed during the sessions. This goal, however, was an overarching goal that they also set in general for their job, not specifically/exclusively for the co-creation sessions.

Identifying participants’ intrinsic motivations or personal benefits was challenging as it was not explicitly addressed during the co-creation sessions. However, personal benefits were explicitly mentioned in the evaluations. Insights were gained, for example, into the problems experienced in the practices of professionals. Furthermore, being able to exchange information with other organizations was seen as valuable. There was a difference between intrinsic and extrinsic motivation for the different participants, with participants from organizations geared towards external motivation to further their organizations’ goals, while the expert-by-experience was driven by personal intrinsic motivation to further the goal of her (and her peers) children being able to take part in sports. The different motivations may have had an effect on the urgency of finding a solution.

Observations indicated that participants derived satisfaction from the collaborative setting where they could engage with peers, discuss their professional work, and enjoy social interactions over tea and cookies. It may be that participants perceived personal benefits in the form of the social gathering as well as making connections in their line of work, which may benefit them later. The sessions were mostly aimed at co-creating solutions, but the evaluations showed participants learned from one another in the exchange of knowledge.

#### 3.2.5. Place and Space

In an ideal situation, the space also provides the grounds for conducting experiments where co-creation takes place. In our case, the spaces for meeting for the sessions were arranged by the participants and were places within their organizations and/or in community centers, allowing for cross-sectoral exchange. "Moreover, the chosen spaces allowed participants to gain insight into one another’s daily working environments, facilitating socio-spatial exchange.

The fact that the researchers stepped into the world of the participating organizations may have signaled that we were not owners of the problem but “merely” there to lead the process. Furthermore, visiting the spaces of different organizations may have influenced the aforementioned dynamics between participants: the locations themselves did not directly influence the process, but they also did not directly link the participants to the theme.

When we expand the concept of space to include the social field, it undoubtedly affects the dynamics. Participants did not have an equal starting position as they differed in life conditions, skills, and experience. For instance, when the expert-by-experience suggested that parents should be able to take their children to a sports club without adhering to any financial regulations, some of the other participants reacted negatively, stating that it was impossible due to the existence of rules. Likewise, the expert-by-experience may have found it difficult to participate fully in conversations on policy regulations around financial aid, as this was not within their area of expertise.

The quality of the social field during these sessions may have also influenced the dynamics, leading to resistance and prolonged discussions on the same topics. A generative social field that brings about change is achieved through shared experiences and participants’ collective efforts to accomplish an outcome. According to the evaluations, this study partially established a generative social field, enabling participants to acquire fresh insights within their domain. Nevertheless, dedicating more time to exploring the potential of collective effort and emphasizing the learning opportunities for individuals could have enhanced the generative impact of the co-creation sessions.

## 4. Discussion

This qualitative study aimed to understand the perspectives of parents and professionals on creating a social environment for low-income families where parents feel safe to request and receive financial support for their children’s sports participation. The study involved co-creation sessions with stakeholders and a parental group interview. 

The study’s results yielded two main conclusions regarding social safety for low-income parents while acquiring financial support for their children’s sports participation. First, parents’ social safety encompassed receiving understandable and consistent information, supportive behavior and knowledge of intermediaries, trustworthy organizations, procedures, and policies based on trust, as well as efficient referral processes that minimized experiencing situations triggering shame and distrust. However, our findings suggest that the reality for parents was quite the opposite. Hence, this study aligns with previous research [13,17] that highlights the numerous obstacles encountered by parents when accessing fee assistance programs. While prior studies have indicated that parents did not perceive stigmatization when utilizing fee assistance programs [13], our findings demonstrate that parents in our sample reported experiencing shame. Our findings underscore how specific barriers faced by parents can contribute to the experience of these emotions. For example, both our study and Tamminen’s study [13] found that parents faced registration issues and sometimes had to wait or return for additional support. However, our research indicates that these circumstances can intensify the feelings of shame experienced by parents since they are required to repeatedly explain their financial needs when seeking support for their children’s sports participation. Identifying these pivotal moments that trigger shame is essential for developing less burdensome and shame-inducing application processes, as argued by Clark (2019) [12]. Additionally, our findings align with the propositions of Social Safety Theory [21] that acquiring (financial) support for their children’s sports participation can pose threats to social safety at various levels, including social interactions within the community (between parents and sports clubs) and at the city level (involving procedures and policies for acquiring financial support, professionals and organizations, and referral processes). As such, in order to increase social safety and, consequently, sports participation by children from low-income families, future social safety interventions may also operate on these different socio-ecological levels. As the sports club seems to be an important entry point for parents in need of financial support to organize their children’s sports participation, it seems especially important to implement the characteristics of a socially safe environment there.

Second, it is concluded that in the stakeholder co-creation sessions, the participants overestimated the level of social safety experienced by parents. Contrary to the assumption that parents would seek support or engage with various stakeholders in their social environment (e.g., social work, education, general practitioner), the parents expressed their preference for solely relying on sports clubs for assistance. This shows that it is vital to include the perspective of low-income parents when developing interventions to increase social safety and, again, to support sport clubs as they are an important entry point for parents who seek support.

In order to address the first aim of how to create a safe social environment for low-income families, a co-creation process with stakeholders was organized. The study’s second aim was to describe this co-creation process, guided by the five elements of co-creation. In relation to purpose, a first conclusion is that participants had different expectations about the purpose of the co-creation sessions. We expected (or hoped) that the purpose would be co-created during the sessions, but based on the recurring questions about purpose, this may not have been accomplished. From this, we advise facilitators of co-creation sessions with participants coming from different organizations to reflect on the purpose and, if necessary, reserve time and attention for co-creating the concrete purpose or desired results of the co-creation session (for example, via the Nominal Group Technique as suggested elsewhere [41]). At the same time, facilitators may want to work on increasing participants’ level of comfort with a temporarily perceived lack of purpose.

Regarding the element of ownership, a second conclusion can be drawn: a clear ownership was not established during the co-creation sessions. Although we expected participants to take ownership of the process and its outputs, given their motivation and affiliation with organizations that could potentially take ownership, the topic of ownership and the transfer of ownership from researchers to participants did not lead to concrete expressions or actions of ownership of solutions. Based on this experience, we recommend co-creation facilitators explicitly address ownership prior to and during the sessions by asking questions such as to what degree participants perceive themselves as responsible for the success of the co-creation process and its outputs and for implementing the co-created solutions. In addition, it is important to clarify the ownership of the co-creation process by asking which organization or stakeholder should assume the role of owner.

Regarding motivation, we first conclude that participants had different types of motivations for joining the co-creation sessions. Personal (intrinsic) motivation played a big part for the expert-by-experience, while professional motivation (extrinsic) was the main driver for organizations. This resulted in different levels of urgency to solve the issue at hand. Consequently, we recommend that future facilitators be mindful of the potential variations in participants’ sense of urgency.

Furthermore, it was observed that stakeholders had distinct professional motivations driven by the goals of their respective organizations. We anticipated that the motivation for the participating organizations would be similar, namely working to resolve the issue of low-income households and access to sports for children. Instead, we found that, for example, one organization was mostly motivated to work on an information package that is supposed to simplify information around financial aid for parents. Another participating party was mostly motivated to make the sports club a one-stop shop for parents. Yet another participant was motivated by resolving the issue but did not have a clear motivation for a certain outcome. It is recommended that future facilitators explicitly address the diverse motivations at the beginning of co-creation sessions and foster transparency regarding the interests of all participants.

In a broader sense, it is advised to allocate ample time and attention to consensus-building during the co-creation process and engage in reflections with participants on the five elements of co-creation.

While the study provides valuable insights into social safety solutions and elements of co-creation, there are some limitations to consider. First, the study included a convenience sample of self-selected stakeholders who voluntarily chose to participate in the co-creation sessions, which may have excluded some relevant stakeholders. Second, only one expert-by-experience represented parents in the stakeholder co-creation sessions due to the other parents’ concerns about social unsafety towards stakeholders coming from organizations. This may have influenced the dynamics and the outputs of the co-creation sessions, given that the expert-by-experience was outnumbered by professionals. However, this limitation raises the question of how to facilitate co-creation sessions with stakeholders who experience a lack of social safety in each other’s presence. Third, the parents who participated in the study were willing and able to talk about their experiences and perspectives related to social safety in Dutch. This may not be representative of all parents’ experiences, particularly those with a relative lack of Dutch language skills or those who do not wish to talk about social safety with researchers. Consequently, the perspectives of parents who have relative lack of Dutch language skills or are hesitant to discuss social safety with researchers may differ from those of the parents in this study when it comes to creating a socially safe environment.

## Figures and Tables

**Table 1:** Elements of co-design as identified by Puerari et al. (2018) [23].

Element	Description
Purpose	Making together (e.g., a product or a service), or Learning together.
Formality	Formal co-creation: structured process with formalized steps, goals, and specific stakeholders. Informal co-creation: less structured, more resistance from participants about method and/or change.
Ownership	Shared ownership: Deliberation and shared consensus on the goal of co-creation are necessary for success. Leadership: Co-creation led by a leader requires less consensus seeking and is easier to adopt.
Motivation/incentives	Intrinsic motivation: co-creating for its own sake, such as learning something new. Extrinsic motivation: co-creating to achieve a desired external outcome, such as meeting a shared goal.
Place/space	Space can facilitate or hinder interaction and stimulate new ideas [31]. Proximity to target groups, peers, or neighborhoods can facilitate learning [32]. The place where qualitative data is gathered can influence the dynamics between researchers and participants [33]. Space also includes the social field, the field of social relations in a group setting (Bordieu (1984), as cited in [34]).

**Table 2:** Description of the sessions.

Session	Activities
Stakeholder co-creation session 1	Introduction
	Discuss the common aim
	Canvas ‘Future Probing’
Stakeholder co-creation session 2	Summary of and reflection on session #1
	Canvas ‘Scenario-Based Action Plan’
Parental group interview	Discuss the output of stakeholder co-creation sessions #1 and #2
Stakeholder co-creation session 3	Summary of and reflection on session #2
	Input from the parental group interview
	Discuss how this input affects the focus of co-creation sessions
Stakeholder co-creation session 4	Summary of and reflection on the previous session
	Co-designed potential solutions in the context of sports clubs
	Canvas ‘Setting Up an Experiment’

## Data Availability

The data presented in this study are available on reasonable request from the corresponding author. The data are not publicly available to protect participants’ privacy.
